# A new knockdown resistance (*kdr*) mutation, F1534L, in the voltage-gated sodium channel of *Aedes aegypti*, co-occurring with F1534C, S989P and V1016G

**DOI:** 10.1186/s13071-020-04201-3

**Published:** 2020-06-29

**Authors:** Raja Babu S. Kushwah, Taranjeet Kaur, Cherry L. Dykes, H. Ravi Kumar, Neera Kapoor, Om P. Singh

**Affiliations:** 1 National Institute of Malaria Research, Sector 8, Dwarka, Delhi, 110077 India; 2 Department of Life Sciences, Jnanabharathi Campus, Bangalore University, Bengaluru, 560056 India; 3 School of Life Sciences, Indira Gandhi National Open University, Maidangarhi, Delhi, 110068 India

**Keywords:** *Aedes aegypti*, Insecticide resistance, Knockdown resistance, Pyrethroid, Voltage-gated sodium channel

## Abstract

**Background:**

*Aedes aegypti* is a primary vector of dengue, chikungunya and Zika infections in India. In the absence of specific drugs or safe and effective vaccines for these infections, their control relies mainly on vector control measures. The emergence of insecticide resistance in vectors, especially against pyrethroids, is a serious threat to the insecticide-based vector control programme. This study reports the presence of multiple knockdown resistance (*kdr*) mutations present in an *Ae. aegypti* population from Bengaluru (India), including a new mutation F1534L.

**Methods:**

*Aedes aegypti* collected from Bengaluru were subjected to insecticide susceptibility tests with DDT, deltamethrin and permethrin. The DNA sequencing of partial domain II, III and IV of the voltage-gated sodium channel (VGSC) was performed to screen *kdr* mutations present in the population and PCR-based assays were developed for their detection. Genotyping of *kdr* mutations was done using PCR-based assays, allelic frequencies were determined, and tests of genetic association of *kdr* mutations with the insecticide resistance phenotype were performed.

**Results:**

The *Ae. aegypti* population was resistant to DDT, deltamethrin and permethrin. The DNA sequencing of the VGSC revealed the presence of four *kdr* mutations, i.e. S989P and V1016G in domain II and two alternative *kdr* mutations F1534C and F1534L in domain III. Allele-specific PCR assays (ASPCR) were developed for the detection of *kdr* mutations S989P and V1016G and an existing PCR-RFLP based strategy was modified for the genotyping of all three known *kdr* mutations in domain III (F1534L, F1534C and T1520I). Genotyping of *Ae. aegypti* samples revealed a moderate frequency of S989P/V1016G (18.27%) and F1534L (17.48%), a relatively high frequency of F1534C (50.61%) and absence of T1520I in the population. Mutations S989P and V1016G were in complete linkage disequilibrium in this population while they were in linkage equilibrium with *kdr* mutations F1534C and F1534L. The alleles F1534C and F1534L are genetically associated with permethrin resistance.

**Conclusions:**

A new *kdr* mutation, F1534L, was found in an *Ae. aegypti* population from Bengaluru (India), co-occurring with the other three mutations S989P, V1016G and F1534C. The findings of a new mutation have implications for insecticide resistance management.
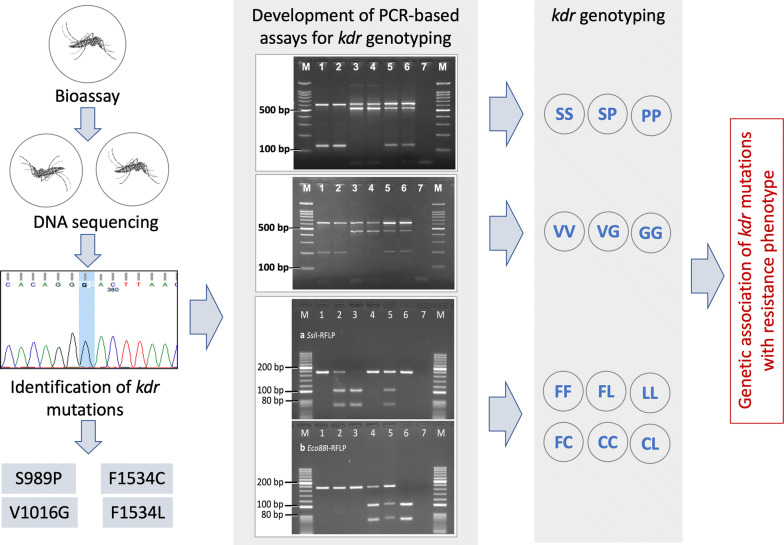

## Background

*Aedes aegypti* is now a widely distributed mosquito species in tropical and subtropical regions and is a primary vector of several human arboviral infections mainly, dengue, chikungunya, yellow fever and Zika viruses. These arboviral infections are increasingly becoming a global health concern due to their rapid geographical spread and high disease burden [1]. The ancestral form of *Ae. aegypti* (*Ae. aegypti formosus*) was found in Africa, which used to feed on non-human primates and after its domestication, *Ae. aegypti* (*Ae. aegypti aegypti*) has now expanded from Africa and colonized most of the pantropical world [2]. During the last few decades, there has been an unprecedented emergence of epidemics of these arboviral diseases [3]. In India, dengue and chikungunya are the main arboviral infections [4–7] with the recent introduction of the Zika virus (ZIKV) [8]. Recently, there had been an outbreak of Zika infections in Jaipur city [9], where *Ae. aegypti* has been incriminated as a Zika vector [10].

Currently, there is no specific drug or safe and effective vaccine available for the control of *Aedes*-borne arboviral infections. For dengue, a live attenuated vaccine, chimeric yellow fever 17D-tetravalent dengue vaccine (CYD-TDV), has been licensed in some countries; however, WHO has not recommended this vaccine for individuals who are seronegative for dengue or who never had dengue infection in the past because this is known to increase the risk of severe dengue in such cases [11]. As such, vector control, including personal protection, is the only effective measure to contain the spread of these arboviral infections, which is primarily based on the use of insecticides and community-engagement for habitat-management [12]. The pyrethroid class of insecticides is of special interest for vector control and is being extensively used in the form of long-lasting insecticidal nets (LLIN), space-spray and as a household repellent. Preference for this insecticide class is mainly due to their low mammalian toxicity, degradability in nature and rapid knockdown effect on insects [13]. However, extensive use of pyrethroids in public health, and also in the agriculture sector, has led to the emergence of resistance against these insecticides in many disease vectors, including *Ae. aegypti.* Several reports of pyrethroid resistance in *Ae. aegypti* have been recorded from different parts of the world [12]. However, such reports were not available from India until 2014. DDT and pyrethroid resistance were reported in Assam and Delhi in 2014 and 2015, respectively [14, 15]. Subsequently, an incipient to moderate level of pyrethroid resistance was reported from West Bengal, India [16, 17].

Understanding the mechanisms of insecticide resistance in vector populations is crucial for effective insecticide resistance management. There are several mechanisms of insecticide resistance, mainly, metabolic resistance where insecticide is detoxified or destroyed at a higher rate than usual, reduced sensitivity of the insecticide-target sites to the insecticide, reduced penetration of the insecticide through cuticle integument and behavioural resistance. One of the known mechanisms of insecticide resistance in mosquitoes against pyrethroids and DDT is knockdown resistance (*kdr*), which is conferred by the alteration in the target site of action, i.e. the voltage-gated sodium channel (VGSC) resulting from non-synonymous mutations. Several *kdr* mutations have been reported in *Ae. aegypti* in different parts of the world, amongst which mutations at three loci, i.e. Ile1011 (I → M/V) and Val1016 (V → G/I) in domain II and Phe1534 (F → C) in domain III (amino acid positions mentioned here and hereafter are based on the sequence of *Musca domestica*, which corresponds to I1018, V1023 and F1565 in *Ae. aegypti* based on GenBank: EU399181), are most commonly reported to be associated with pyrethroid resistance [15, 18–24]. Functional expression analysis of VGSC in *Xenopus* oocyte confirmed the role of a total of four mutations *viz* V410L [25], I1011M, V1016G and F1534C [26] in reducing the sensitivity of VGSC to pyrethroids. Mutations S989P and D1794Y found in association with V1016G neither impose additive or synergistic effects [26] but may have a compensatory role to overcome fitness cost associated with V1016G. The mutation T1520I, found in India, which was initially thought to be a compensatory mutation to F1534C, [15] was later found to have an additive effect on the sensitivity of VGSC [27]. The presence of such *kdr* mutations in the Indian subcontinent has been screened only in a northern Indian population [15] and an eastern Indian population [28]. To increase knowledge of the spatial distribution of the *kdr* mutations in India, we screened a southern Indian population (Bengaluru metropolitan city) of *Ae. aegypti* and report the presence of a new mutation F1534L, co-occurring with mutations F1534C, S989P and V1016G and their association with insecticide resistance. This is the first report of the presence of F1534L mutation in *Ae. aegypti*.

## Methods

### Mosquito collection

Immatures (larvae and pupae) of *Aedes* sp. were collected from domestic and peri-domestic breeding sites from the Basavanagudi area of Bengaluru city (77° 56–57′ E, 12° 92–95′ N) during October-November 2014 and March-April 2015. Oral informed consent was obtained from the owners of the houses for the collection of immatures from the residential premises. Immatures were reared in the laboratory until their emergence into adults (F_0_) and identified using morphological characters. F_1_ progenies were also obtained. To get F_1_ progenies, approximately 50 freshly emergent male and female mosquitoes were pooled in a mosquito cage and kept in an insectary maintained at a temperature of 27 ± 1°C, relative humidity (RH) 60–70% and a photoperiod of 14:10 h (light:dark) ratio. Mosquitoes were provided access to 10% glucose-soaked cotton pads. On the fourth day of emergence, female mosquitoes were fed on chicken blood through an artificial membrane (Parafilm®; Bemis NA, Neenah, USA) and a single batch of eggs was obtained after 72 h of blood-feeding. Eggs were allowed to hatch in water and larvae were reared in enamel trays supplemented with fish-food until pupation. Pupae were removed from the trays and placed in a bowl containing water and kept inside an insect cage (measuring 30 × 30 × 30 cm) for emergence into adults. Adults were fed on 10% glucose-soaked cotton pads.

### Exposure of insecticide to mosquitoes (bioassay)

Three-four-day-old adult *Ae. aegypti* female mosquitoes were exposed to 0.05% deltamethrin-, 0.75% permethrin- or 4% DDT-impregnated papers (supplied by the WHO collaborative centre, Vector Control Research, Universiti Sains, Malaysia) for 1 h, following the WHO standard insecticide-susceptibility test guidelines for malaria vectors [29] in absence of WHO recommended discriminatory dose for *Aedes* mosquitoes at the time of the bioassay, which is currently 0.03% and 0.25% for deltamethrin and permethrin, respectively [30]. Following exposure of insecticide, mosquitoes were transferred to recovery tubes and mortalities were recorded after 24 h of recovery. During recovery, a pad of a cotton wool soaked in 10% glucose water was placed on the mesh-screen end of the holding tubes. Individual dead and alive mosquitoes were kept in 1.5 ml microcentrifuge tubes with a piece of silica gel for DNA isolation and stored at − 20 °C. All bioassays were carried out in a laboratory maintained at 25 °C with a RH of 60–70%. A total of 572 field-collected F_0_ and 242 F_1_ mosquitoes were exposed to insecticide papers, alongside controls.

### DNA isolation and sequencing

DNA from individual mosquitoes was isolated following the method described by Livak et al. [31], after removing one third of the posterior abdomen that carries spermatheca (to avoid contamination of sperm from the male mating partner) and stored at 4 °C. Some of the mosquitoes were sequenced for partial domains II, III and IV of the VGSC. Primers used for amplification of domains II, III and IV are shown in Table 1. A common PCR protocol and PCR conditions were used for amplification of partial domains II, III and IV of the VGSC. The PCR reaction (25 μl) contained 1× buffer, 1.5 mM MgCl_2_, 200 µM of each dNTP, 0.2 µM of each primer and 0.625 units of *Taq* polymerase (AmpliTaq Gold; Applied Biosystems, Foster City, USA). The PCR conditions were: an initial denaturation at 95 °C for 3 min, followed by 35 cycles each of denaturation at 95 °C for 15 s, annealing at 55 °C for 15 s and extension at 72 °C for 30 s, followed by a final extension step at 72 °C for 7 min. PCR products were purified using ExoSAP-IT PCR Product Cleanup (Exonuclease I—shrimp alkaline phosphatase; Applied Biosystems) and were either sent to Macrogen Inc. (Seoul, South Korea) for sequencing or sequenced in an in-house sequencing facility (ABI Prism 3730xl; Applied Biosystem, Foster City, USA). The sequence chromatograms were edited using Finch TV ver. 1.5.0 (http://www.geospiza.com/). Sequencing was performed from both directions of the DNA strands. The numbers of individuals successfully sequenced were 294 samples for domain II, 27 samples for domain III and 12 samples for domain IV.

**Table 1:** List of the primers used in this study

Primers used for	Name of primer	Nucleotide sequence (5′–3′)	Reference
Amplification and DNA sequencing of domain II	AedIIF	AGACAATGTGGATCGCTTCC	This study
	AedIIR	GGACGCAATCTGGCTTGTTA	This study
	IIP_F	GGTGGAACTTCACCGACTTC	Yanola et al. [43]
	IIS6_R	GGACGCAATCTGGCTTGTTA	Yanola et al. [43]
Amplification and DNA sequencing of domain III	AedIIIF	AAC GTT CAA GGG CTG GAT CC	This study
	AedIIIR	TTC GAG CCC ATC TTT TTC AT	This study
Amplification and DNA sequencing of domain IV	5380F1	CCAAAGGTATCCGAACGTTGCTG	Chang et al. [36]
	5380R1	CCGATCATCAACTCACCAGAAAC	Chang et al. [36]
Allele-specific PCR for S989P	AedIIF	AGACAATGTGGATCGCTTCC	This study
	AedIIR	GGACGCAATCTGGCTTGTTA	This study
	PPF	GGCGAGTGGATCGAAC	This study
	SSR	GCATACAATCCCACATGGA	This study
Allele-specific PCR for V1016G	AedIIF	AGACAATGTGGATCGCTTCC	This study
	AedIIR	GGACGCAATCTGGCTTGTTA	This study
	VVF	TCCCACTCGCACAGGT	This study
	GGR	GGCTAAGAAAAGGTTAAGTC	This study
PCR-RFLP for F1534C/L and T1520I	AekdrF	TGGGAAAGCAGCCGATTC	Kushwah et al. [15]
	AekdrR	CCTCCGTCATGAACATTTCC	Kushwah et al. [15]

### Cloning and sequencing

It was not possible to identify the correct amino acid codon through DNA sequencing in samples that were found heterozygous for two nucleotide base positions, i.e. first and second codons of the F1534 residue. Therefore, we cloned and sequenced five such heterozygous samples to identify the correct codon. PCR products were amplified using primers AekdrF and AekdrR with high fidelity *Taq* DNA polymerase (Phusion® High-Fidelity DNA Polymerase; Thermo Fisher Scientific, Waltham, USA). The PCR conditions were: initial denaturation at 95 °C for 3 min, followed by 35 cycles each with a denaturation step at 95 °C for 30 s and an annealing/extension step at 68 °C for 1 min, followed by a final extension step at 72 °C for 10 min. The PCR product was purified using QIAquick PCR Purification Kit (Qiagen, Hilden, Germany) and incubated at 72 °C for 10 min in a reaction mixture containing 200 μM of dATP, 1.5 mM MgCl_2_, 0.625 units of *Taq* polymerase and 1× buffer, in order to add A-tail. Finally, the PCR product was purified and cloned in pGEM-T Easy Vector System (Promega, Madison, Wisconsin, USA) as per the manufacturer’s protocol. Chemically competent *Escherichia coli* cells (DH5α) were transformed with the recombinant DNA and grown on a LB-Agar plate supplemented with 100 μg/ml ampicillin. Positive clones were selected by blue/white screening and PCR-amplified using universal primers SP6 and T7. The PCR products of individual clones were sequenced at Macrogen (Seoul, South Korea), using primers SP6 and T7. Sequences were aligned using MEGA5 [32]. A total of 15 clones were sequenced.

### Genotyping of domain II *kdr* alleles (S989P and V1016G)

**Fig. 1:**
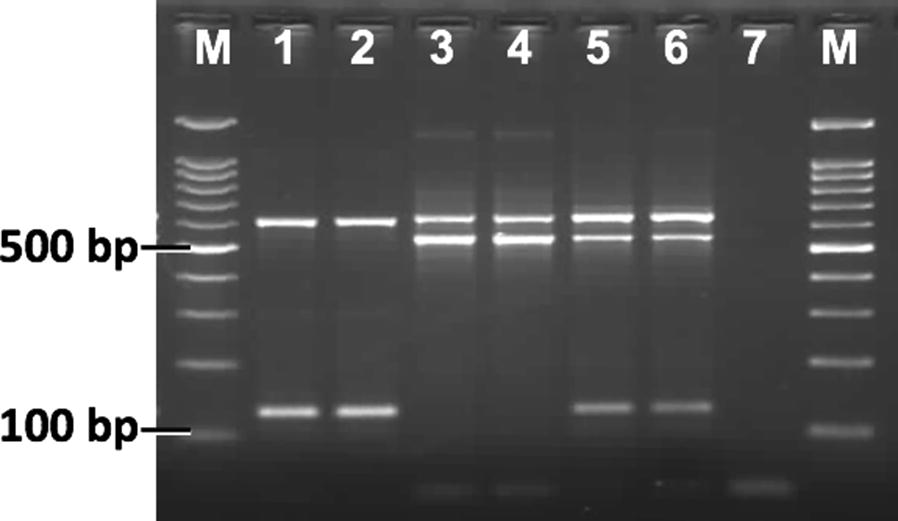
Gel photographs of allele-specific PCR for the identification of S989-*kdr* alleles. Lane M: 100 bp ladder; Lanes 1, 2: SS; Lanes 3, 4: PP; Lanes 5, 6: SP; Lane 7: negative control

**Fig. 2:**
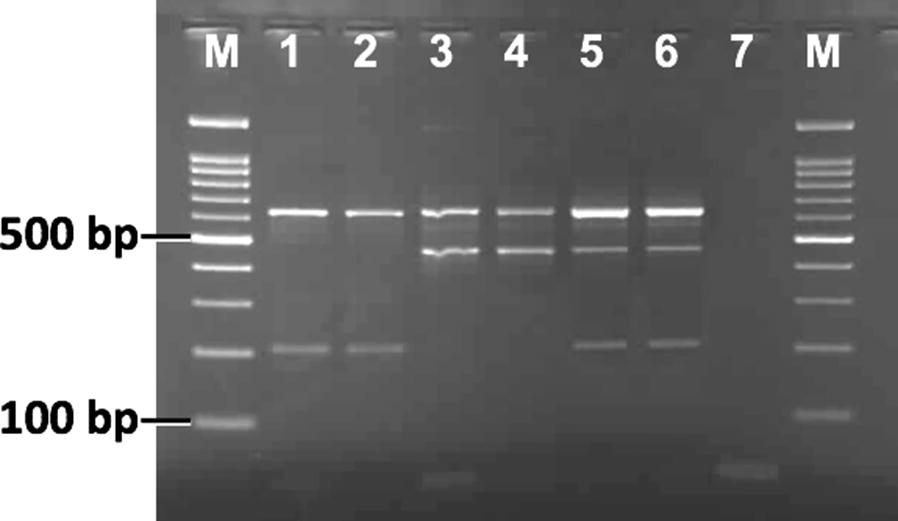
Gel photographs of allele-specific PCR for the identification of V1016-*kdr* alleles. Lane M: 100 bp ladder; Lanes 1, 2: VV; Lanes 3, 4: GG; Lanes 5, 6: VG; Lane 7: negative control

For genotyping of *kdr* alleles at residue S989 and V1016, separate allele-specific PCR assays were designed because the existing PCR developed by Li et al. [33] exhibited null alleles, in most cases. The list of primers used for allele-specific PCR (ASPCR) is shown in Table 1. The PCR conditions for both PCRs were identical. The PCR was carried out using DreamTaq® Hot Start DNA polymerase ready reaction mixture (Thermo Fisher Scientific). The thermal cycling conditions of PCR were as follows: initial denaturation at 95 °C for 3 min, followed by 20 cycles, each of denaturation at 95 °C for 15 s, annealing for 30 s at temperature from 65 °C to 45 °C with an decrement of 1 °C each cycle and extension at 72 °C for 60 s. In the next 15 cycles, the cycling conditions were similar except the annealing temperature was kept constant at 45 °C. This was followed by a final extension step at 72 °C for 10 min. The primers used for the S989-ASPCR were 1.0 µM of AedIIF, 1.5 µM of AedIIR, 0.6 µM of SSR and 0.45 µM of PPF. For the V1016-ASPCR, primers used were 1.2 µM of AedIIF, 2.25 µM of AedIIR, 0.45 µM of VVF and 1.2 µM of GGR. The sizes of the universal amplicons are 620 or 636 bp for both ASPCRs (variation is due to indels in the intron). The sizes of Ser (S)- and Pro (P)-specific amplicons are 129 bp and 525/549 bp, respectively, for the S989-ASPCR. For the V1016-ASPCR, sizes of Val (V)- and Gly (G)-specific amplicons are 209 bp and 446/462 bp, respectively. PCR products were electrophoresed on a 2.0% agarose gel containing ethidium bromide and visualized under UV using a gel documentation system (Figs. 1 and 2).

### Genotyping of domain III *kdr* alleles (T1520I, F1534C and F1534L)

**Fig. 3:**
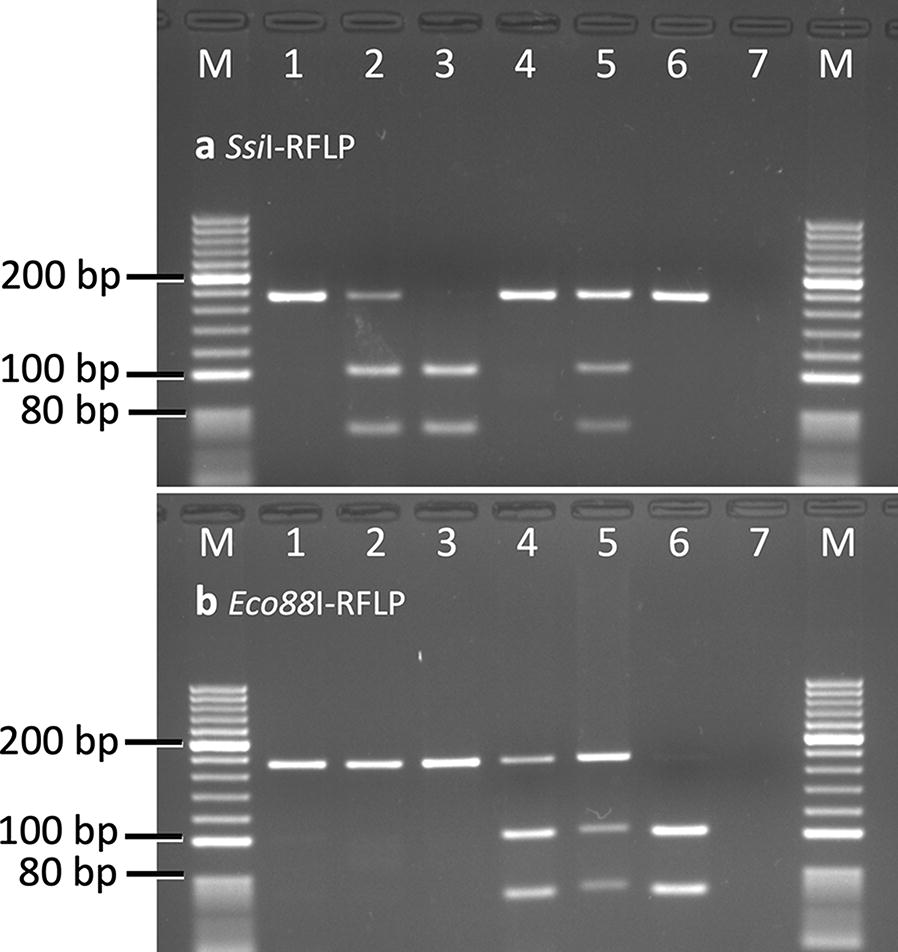
Gel photographs of **a***Ssi*I- and **b***Eco88*I-PCR-RFLP digests showing the banding pattern of the different F1534-*kdr* genotypes. Scoring of genotypes are done based on bands present in both *Ssi*I- as well as *Eco88*I-PCR-RFLP following criteria defined in Table 3. Lane M: 20 bp ladder; Lane 1: FF; Lane 2: FC; Lane 3: CC; Lane 4: FL; Lane 5: LC; Lane 6: LL; Lane 7: negative control

Previously, Kushwah et al. [15] reported a PCR-RFLP for the genotyping of T1520I and F1534C, where a single PCR product amplified using primers AekdrF and AekdrR was subjected to RFLP with two restriction enzymes *BsaB*I (for T1520I) and *Ssi*I (for F1534C) in separate tubes. To include RFLP for the identification of the new mutation F1534L using the same amplicon, we searched for the F1534L-specific restriction enzyme site using an online tool available at http://insilico.ehu.es/restriction/two_seq. We found a unique restriction site *Eco88*I in the DNA sequence for F1534L and therefore, for the genotyping of all *kdr* alleles present in domain III, the PCR-RFLP method was modified. In the modified procedure, an additional restriction-digestion process was performed in a separate tube with restriction enzyme *Eco88*I for the identification of the new mutation F1534L. The additional restriction-digestion reaction mixture (20 μl) contained 5 μl of PCR-amplified product, 2 units of *Eco88*I enzyme and 1× buffer (Thermo Fisher Scientific). This was incubated for 4 h or overnight at 37 °C followed by electrophoresis on 2.5% agarose gel. The RFLP products were visualized in a gel documentation system (Fig. 3). The criteria for the scoring of F1534 alleles were modified (see Table 2), while the criterion for scoring the T1520 allele remained unchanged [15].

**Table 2:** Criteria for the scoring of F1534 alleles in PCR-RFLP assays

F1534 genotype	Size of PCR-RFLP bands in	
	*Eco88*I-RFLP (bp)	*Ssi*I-RFLP (bp)
FF	171	171
FC	171	171, 103 and 68
FL	171, 103 and 68	171
CC	171	103 and 68
CL	171, 103 and 68	171, 103 and 68
LL	103 and 68	171

### Statistical analyses

Hardy–Weinberg equilibrium (HWE) of *kdr*-alleles in a population was tested using Fisher’s exact test. Phasing of haplotypes and estimation of their frequencies were performed using Arlequin 3.5 software [34]. Association of *kdr* genotypes with resistance phenotype was estimated using Fisher’s exact test and odds ratio (OR). Association of haplotypes with insecticide resistance phenotypes was performed using the additive model [35] and the test of significance was carried out using Fisher’s exact test and odds ratio (OR). The Wright’s inbreeding coefficient was calculated using formula F = (He − Ho)/He, where ‘He’ is expected heterozygosity and ‘Ho’ is observed heterozygosity.

## Results

### Insecticide-bioassay

The percent corrected mortalities of adult female *Ae. aegypti* after 1 h exposure and 24 h recovery against WHO’s diagnostic insecticide papers (recommended for determining the susceptibility of *Anopheles* mosquitoes) is shown in Table 3. The mortalities in the F_0_ mosquitoes were 5–8% for 4% DDT, 20–22% for 0.75% permethrin (PER) and 18–24% for 0.05% deltamethrin (DEL). The mortalities in F_1_ progeny for permethrin and deltamethrin were 36% and 30%, respectively. There were no significant differences in percent mortalities of F_0_ mosquitoes during different collections.

**Table 3:** Results of insecticide-bioassay of *Ae. aegypti*

Insecticide paper (concentration)	Month and year (filial generation)	No. of mosquitoes exposed (*n*)	% corrected mortality^a^ (95% CI)
DDT (4%)	March 2015 (F_0_)	60	5.0 (1.70–13.70)
	April 2015 (F_0_)	143	9.09 (5.39–14.39)
Permethrin (0.75%)	November 2014 (F_0_)	74	20.27 (12.69–30.79)
	April 2015 (F_0_)	106	21.70 (14.92–30.46)
	April 2015 (F_1_)	92	35.87 (26.82–46.06)
Deltamethrin (0.05%)	November 2014 (F_0_)	57	17.54 (9.82–29.36)
	April 2015 (F_0_)	75	24.00 (15.75–34.78)
	April 2015 (F_1_)	150	30.00 (23.24–37.76)

^a^No mortality was recorded in the controls

### Identification of *kdr* mutations in *Ae. aegypti* population

The DNA sequencing of partial domain II, III and IV of the VGSC of the *Ae*. *aegypti* population collected from Bengaluru, India, revealed the presence of four mutations, i.e. S989P and V1016G in domain II, and F1534C and F1534L in domain III. No mutation was found in domain IV. To our knowledge, the mutation F1534L is being reported for the first time in *Ae. aegypti.* Mutations S989P and V1016G present in domain II were due to substitution on the first base of the codon (TCC → CCC) and second base of the codon (GTA → GGA), respectively. In most of the cases, the identification of S989 and V1016 mutations were based on 1× sequencing data, where the forward sequence was used for identification of S989 alleles and the reverse sequence was used for V1016 alleles. This was due to the presence of an ambiguous sequence in the downstream sequence resulting from multiple indels present in the intron between these two *kdr* loci. In the course of this study, a total of 294 samples were sequenced for partial domain II of which 178 were homozygous wild-type for both residues (SS at residue S989 and VV at residue V1016), 92 were heterozygous (SP and VG) and 24 were mutant homozygous (PP and GG). The other two alternative mutations F1534L and F1534C present in domain III were due to T > C substitution on the first position of the codon, leading to Phe (TTC) → Leu (CTC) mutation, and T > G substitution on the second position of the codon leading to Phe (TTC) → Cys (TGC) mutations, respectively. Out of the 27 individuals sequenced for domain III, 1 was homozygous wild-type for Phe (TTC) at residue F1534, 7 were homozygotes for Cys (TGC), 4 were homozygotes for Leu (CTC), 4 samples were heterozygotes for each of Phe/Cys and Phe/Leu and 7 had mixed bases at the first and second position of the codon, i.e. with YKC, which could be either heterozygote for Leu/Cys (CTC + TGC) or Phe/Arg (TTC + CGC). The latter combination was ruled out as sequencing of 15 cloned PCR products from 5 heterozygote samples having sequence YKC revealed the presence of 2 haplotypes, one with CTC (Leu) and another with TGC (Cys). We also observed that the haplotype with the F1534L mutation had a restriction site for *Eco88*I (5′-C↓YCGRG-3′). Therefore, all 7 heterozygote samples with the sequence YKC were also subjected to PCR-RFLP with *Eco88*I and all were partially cleaved, indicating the presence of Leu/Cys (CTC + TGC) heterozygote. The DNA sequencing of 12 samples for partial domain IV revealed the absence of any non-synonymous mutation, including D1794Y reported elsewhere [36]. Additionally, 25 samples were checked for the presence of the D1794Y mutation using PCR-RFLP following Chang et al. [36] and none were found to have this mutation.

### Development of PCR-based assay for genotyping of *kdr* alleles

For genotyping of F1534-*kdr* alleles, we modified the PCR-RFLP developed by Kushwah et al. [15] where an additional restriction enzyme *Eco88*I was used for the identification of the new allele F1534L. For genotyping of S989- and V1016-*kdr* alleles, allele-specific PCRs (ASPCR) were developed for each locus. The genotyping results from these methods were validated through DNA sequencing. The genotyping result of 27 samples that were sequenced for partial domain III of the VGSC (FF = 1, CC = 7, LL = 4, FL = 4, FC = 4 and LC = 7) and 294 samples sequenced for domain II (SS/VV = 178, PP/GG = 24, SP/VG = 92) matched with DNA sequencing results.

### Genotyping of *kdr* alleles and their linkage equilibrium

Genotyping results of *kdr* alleles at loci F1534, S989 and V1016 carried out on 572 field-collected F_0_ populations are shown in Table 4. The overall frequencies of F1534 (F), F1534C (C), F1534L (L), S989 (S), S989P (P), V1016 (V) and V1016G (G) were 0.32, 0.51, 0.17, 0.82, 0.18, 0.82 and 0.18, respectively, in collections carried out in year 2014 and 2015. The mutation T1520I was absent in this population. It was observed that the frequency of observed F1534-*kdr* genotypes is not in Hardy–Weinberg equilibrium (HWE), while S989 and V1016-*kdr* genotypes were in HWE in pooled samples. Analysis of the inbreeding coefficient for F1534-*kdr* alleles revealed a positive value indicating the presence of fewer heterozygotes than expected (*F* = 0.09). Additionally, a total of 242 F_1_ individuals were also genotyped for *kdr* alleles. Thus, a total of 814 samples (F_0_ and F_1_ combined) were genotyped for *kdr* alleles present in domains II and III. The frequency of individuals with different genotype combinations at three loci is shown in Additional file 1: Table S1. Estimation of the gametic phase based on Gibbs sampling strategy implemented in Arlequin 3.5 [34] revealed the presence of only 4 haplotypes namely, SVF (wild), SV**C**, SV**L** and **PG**F (mutant alleles are shown in bold letters) with allelic frequencies of 0.176, 0.518, 0.158 and 0.147, respectively (Additional file 2: Text S1). This indicates that two *kdr* mutations in domain II, i.e. S989P and V1016G, are in linkage disequilibrium but were in linkage equilibrium with mutant *kdr*-alleles in domain III (C and L). Thus, *kdr* mutations in domain II (S989P and V1016G) were not found with any of the *kdr* mutations present in domain III (F1534C or F1534L) on the same haplotype.

**Table 4:** Result of *kdr* genotyping of *Ae. aegypti* in field population (F_0_): distribution of *kdr* genotypes, allelic frequencies and compliance to Hardy–Weinberg equilibrium

Collection date	*n*	*kdr* genotypes												Allele frequencies							*P*_HWE_		
		F1534						S989			V1016			F1534			S989		V1016		F1534	S989	V1016
		FF	FC	CC	FL	LL	LC	SS	SP	PP	VV	VG	GG	F	C	L	S	P	V	G			
October–November 2014	148	12	26	20	20	8	62	120	20	8	120	20	8	0.24	0.43	0.33	0.88	0.12	0.88	0.12	0.00	0.00	0.00
March–April 2015	424	61	151	110	22	0	80	270	135	19	270	135	19	0.35	0.53	0.12	0.80	0.20	0.80	0.20	0.00	0.92	0.92
Total	572	73	177	130	42	8	142	390	155	27	390	155	27	0.32	0.51	0.17	0.82	0.18	0.82	0.18	0.00	0.09	0.09

*Abbreviation*: *P*_HWE_, test of significance for the deviation from Hardy–Weinberg equilibrium

### Genetic association of *kdr* with phenotypic insecticide resistance

The distribution of individuals with different *kdr* genotypes in respect to F1534, S989 and V1016 loci in dead and alive mosquitoes (F_0_ and F_1_) after exposure to 0.75% permethrin (type I pyrethroid), 0.05% deltamethrin (type II pyrethroid) and 4% DDT is shown in Table 5. With the present data, testing the genotypic association of *kdr* mutations with the insecticide resistance phenotype was constrained due to very low frequency (0.04) of the wild-type form (with respect to all the three *kdr* loci, i.e. SSVVFF). Statistical analyses were performed to test the association of *kdr* genotypes with resistance in each insecticide-treatment group on pooled samples. A low level of association of *kdr-*genotypes could be established, only in cases with higher frequencies, where genotypes SSVV**CC** and SSVV**LC** were significantly associated with DDT and permethrin resistance and SPV**G**F**C** and SPV**G**F**L** were associated with permethrin resistance (Table 5). No association could be established with genotypes present in low frequencies.

**Table 5:** Distribution of *kdr* genotype-combinations with respect to locus S989, V1016 and F1534 in dead and alive mosquitoes (after 1 h exposure and 24 h recovery period) and their genetic association with insecticide resistance phenotype

Insecticide	Dead/alive	Statistical tests	Genotypes with respect to S989, V1016 and F1534									
			SSVVFF	SSVVFC	SSVVFL	SSVVCC	SSVVLL	SSVVLC	SPVGFF	SPVGFC	SPVGFL	PPGGFF
DDT 4%	Alive		3	36	2	46	0	21	16	48	7	8
	Dead		2	8	0	0	0	0	1	3	1	1
		Fisher’s exact	–	ns	nd	*P *< 0.01	nd	nd	nd	ns	nd	nd
		OR (95% CI)	–	3.00 (0.43–21.01)	nd	Infinity	nd	nd	nd	10.67 (1.26–90.29)	nd	nd
PER 0.75%	Alive		2	15	6	79	10	63	1	24	13	11
	Dead		4	27	3	16	3	15	8	4	2	3
		Fisher’s exact	–	ns	nd	*P *< 0.05	nd	*P *< 0.05	nd	*P *< 0.05	nd	nd
		OR (95% CI)	–	1.11 (0.18–6.80)	nd	9.87 (1.66–55.58)	nd	8.40 (1.4–50.22)	nd	12.00 (1.62–88.71)	nd	nd
DEL 0.05%	Alive		10	38	19	52	4	48	11	21	5	5
	Dead		8	23	7	23	0	10	8	8	1	1
		Fisher’s exact test	–	ns	ns	ns	nd	*P *< 0.05	ns	ns	nd	nd
		OR (95% CI)	–	1.32 (0.46.3.83)	2.17 (0.60–7.74)	1.81 (0.63–5.17)	nd	3.84 (1.21–12.16)	1.10 (0.30–4.04)	2.10 (0.61–7.23)	nd	nd

*Abbreviations*: OR, odds ratio; CI, confidence interval; DDT, dichloro diphenyl trichloroethane; PER, permethrin; DEL, deltamethrin; ns, not significant; nd, not determined

Due to the constraint associated with the genotypic association, the allelic association of different mutant haplotype-alleles with resistance phenotype was tested using an ‘additive model’ [35]. In this study, it was noted that there are just four distinct gametic phases (SVF, SV**C**, SV**L** and **PG**F) that are being inherited as a single unit without any evidence of recombination. Therefore, the test of association of the three different haplotype-alleles (having mutant allele/s) with insecticide resistance was done. Although similar association can be done with SNP-alleles, in that case, allele ‘F’ from haplotype SVF and **PG**F will be weighted equally in the test of genetic association beside the fact that former haplotype is wild-type in respect to all three loci and the latter is linked to two *kdr* mutations (**P** and **G**). The distribution of haplotype-alleles in dead and alive mosquitoes following insecticide bioassay and results of tests of the genetic association is shown in Table 6. The result shows that all the haplotype bearing mutant alleles (SV**C**, SV**L** and **PG**F) are significantly associated with permethrin resistance with a high value of significance (*P *< 0.0001). Besides, SV**C** and SV**L** were associated with DDT and deltamethrin resistance, respectively, with a low level of significance. (*P *< 0.01).

**Table 6:** Distribution of *kdr* haplotype-alleles in dead and alive mosquitoes after exposure to the insecticide papers (1 h exposure followed by 24 h recovery) and their genetic association with insecticide resistance phenotype

Insecticide	Dead/alive	Haplotype-allele counts				Fisher’s exact test			OR (95% CI)		
		SVF	SVC	SVL	PGF	SVF *vs* SVC	SVF *vs* SVL	SVF *vs*PGF	SVF *vs* SVC	SVF *vs* SVL	SVF *vs*PGF
DDT 4%	Alive	60	197	30	87	*P *< 0.01	ns	ns	3.88 (1.65–9.11)	6.50 (0.81–52.06)	2.69 (1.01–7.15)
	Dead	13	11	1	7						
PER 0.75%	Alive	26	260	102	60	*P *< 0.0001	*P *< 0.0001	*P *< 0.0001	5.90 (3.40–10.15)	6.94 (3.64–13.24)	5.31 (2.64–10.67)
	Dead	46	78	26	20						
DEL 0.05%	Alive	88	211	80	47	ns	*P *< 0.01	ns	1.49 (0.98–2.27)	2.73 (1.48–5.04)	1.52 (0.81–2.85)
	Dead	54	87	18	19						

*Abbreviations*: OR, odds ratio; CI, confidence interval; DDT, dichloro diphenyl trichloroethane; PER, permethrin; DEL, deltamethrin; ns, not significant

## Discussion

Insecticide resistance is rapidly increasing in Indian *Ae. aegypti* populations. Resistance to DDT and pyrethroids in adult *Ae. aegypti* in India was reported as early as in the year 2014 in Assam [14] and the year 2015 in Delhi [15]. Subsequently, incipient to moderate levels of resistance were reported in West Bengal [16, 17]. The present study reports a relatively higher degree of resistance to both DDT and pyrethroids as compared to previous reports. However, the bioassays in the above-cited studies were carried out using WHO’s discriminatory doses of insecticides recommended for malaria vectors, which is much higher than the discriminatory dose recommended for *Aedes* mosquitoes at least for pyrethroids [30]. At the time of this study, the discriminatory doses for *Aedes* were not available. Thus, the resistance against pyrethroids in the study population is assumed to be much higher than as estimated. High level of resistance in Bengaluru metropolitan city against DDT and pyrethroids are intriguing because the extensive use of these insecticides are limited to rural areas, where they are used in the form of insecticide residual spray (IRS), besides the use of pyrethroid-impregnated long-lasting insecticidal nets (LLINs). In urban areas, IRS is not used and the use of insecticides is limited to space-spraying and use of pyrethroid-based household anti-mosquito gadgets (liquid vaporizer, mats and coils) in the community for the personal protection against mosquito nuisance.

Understanding of the mechanisms of insecticide resistance is crucial to any insecticide-based disease-vector control programme. There are several mechanisms of insecticide resistance, mainly, metabolic resistance and reduced target site sensitivity. Reduced sensitivity of VGSC, the target site for DDT and pyrethroids, in insects is due to conformational changes in the VGSC arising from one or more mutations, commonly referred to as knockdown resistance (*kdr*) mutation. In *Ae. aegypti*, several mutations have been identified at nine different loci [12]. Among these, F1534C, S989P and V1016G are widely-reported *kdr* mutations and have been found associated with resistance against DDT and pyrethroids [37–39]. Limited studies in the Indian *Ae. aegypti* revealed the presence of F1534C and a novel mutation T1520I (linked to F1534C) in Delhi (northern Indian population) [15] and F1534C, V1016G and T1520I in West Bengal (eastern Indian population) [28]. The presence of S989P was not investigated in the study carried out in the West Bengal population. The present study carried out in Bengaluru (southern India) revealed the presence of four *kdr* mutations in *Ae. aegypti*, i.e. S989P, V1016G, F1534C and F1534L. In a previous study carried out in the Delhi population in 2014 [15], we did not find the three mutations reported in the present study, i.e. S989P, V1016G and F1534L. Similarly, a novel mutation T1520I reported in Delhi was absent from Bengaluru. To ensure that we did not miss the detection of the other three mutations (S989P, V1016G and F1534L) in the Delhi population during the previous study, we genotyped 184 *Ae. aegypti* samples collected from Delhi in August 2018 and did not find any of these three mutations in the Delhi population. Thus, there is a contrasting difference in the distribution of *kdr* alleles in two different geographical locations, which are approximately 1700 km apart. In another part of India (West Bengal, eastern India), three mutations, i.e. F1534C, T1520I and V1016G were reported but the presence of S989P was not investigated in this study.

To the best of our knowledge, this study reports for the first time the presence of F1534L in *Ae. aegypti*, although this mutation has been reported in another closely related aedine mosquito *Ae. albopictus* [40, 41]. The residue F1534 appears to be important from a knockdown resistance point of view, and so far, three alternative mutations have been reported at this residue in aedine mosquitoes, i.e. F1534C, F1534L and F1534S. The latter has been reported to be present in 12 populations of *Ae. albopictus* across Asia, Africa, America and Europe, and found to be associated with deltamethrin resistance [42]. However, so far, no such mutation is reported in *Ae*. *aegypti*. The discovery of a new *kdr* mutation (F1534L) is of global concern and needs to be investigated in other parts of the world. For genotyping of the field population, other available PCR methods used for F1534-*kdr* genotyping either need to be modified to include F1534L or the method described in this communication is to be followed. Theoretically, other existing PCR assays developed for genotyping of F1534*-kdr* [43, 44] will identify F1534L as a wild genotype or will provide a null allele.

For monitoring *kdr* mutations in field conditions, we developed a highly specific PCR-RFLP-based assay for simultaneous detection of all the three mutations (five alleles in total) reported in domain III-S6 of the VGSC at locus T1520 and F1534. The PCR-RFLP for the identification of all five alleles is advantageous over other PCR-based methods being highly specific due to the high sequence-specificity of restriction enzymes. Additionally, in this PCR-RFLP assay, unlike other assays, a single PCR product is required for genotyping of all five alleles present at loci T1520 and F1534. For genotyping of mutations present in domain II-S6, we developed two allele-specific PCR (ASPCR) assays, one each for S989P and V1016G mutations, because available PCR based methods were either for V1016G only [45] or two independent PCRs were required to be performed for each locus [33]. Moreover, in the latter case, at least one primer for each PCR assay was designed from intron regions, which are highly polymorphic and can result in null alleles. Our ASPCRs for genotyping of S989P and V1016G alleles are advantageous over other available PCR assays for these mutations because our method needs a single assay for each locus. In our PCR assays, flanking primers are common for both PCRs. However, being based on single base mismatch, all ASPCR are prone to non-specific extension and a high degree of optimization is required. ASPCR is sensitive to a change in the type of reagents and PCR thermal cycling conditions. Discrepancies were noticed with such PCR-based genotyping of F1534 *kdr-*alleles in an earlier study [43].

Although several *kdr* mutations have been reported in *Ae. aegypti*, the most frequently reported mutations (V1016G and F1534C) are known to significantly reduce the sensitivity of VGSC to pyrethroids in a functional expression study carried out in a *Xenopus* oocyte [26]. In the present study, allelic association shows that haplotypes bearing V1016G (along with S989P), F1534C and F1534L are associated with insecticide resistance. The allelic association-study presented here is constrained due to the presence of multiple mutations present in the population. A significant number of F1534 alleles (C and L) were from heterozygotes 1534C/L (haplotype SV**C**/SV**L**), where the frequency of 1534C was higher than that of 1534L and the inferred effect of 1534L could be due to 1534C alleles present in C/L heterozygotes. To testify if there is a possible influence of 1534C on 1534L in the analysis due to C/L heterozygotes, in another analysis, we excluded the data of C/L heterozygotes from the association test and found that the 1534L mutation is still strongly associated with at least permethrin resistance (OR: 6.27, 95% CI: 2.75–14.30, *P* < 0.0001). The genetic association studies presented here are based on the assumption that none of the other insecticide-resistance mechanisms are linked to *kdr* alleles and are distributed randomly in the test groups. Further, this analysis is based on the assumption that *kdr* alleles have an additive effect on the trait. Confirmation of the putative role of F1534L on the insecticide resistance phenotype is warranted using genetic association involving homozygous wild and mutant mosquitoes (which were present in low frequency in this study) or functional expression of this mutation on the sensitivity of the VGSC to the insecticides.

The co-occurrence of F1534C with S989P and V1016G may be of serious concern if all three mutations are present on the same haplotype. It has been shown through site-directed mutagenesis that such a combination (S989P + V1016G + F1534C) may result in a seriously high degree of resistance against permethrin as well as deltamethrin (1100- and 90-fold, respectively) [46]. In the present study, although we found mutations S989P/V1016G and F1534C/F1534L together in an individual mosquito, they were always in a heterozygous condition. Phasing out of haplotypes revealed that there are just four haplotypes SVF, PGF, SVL and SVC present in this population. Thus, S989P and V1016G are always found on the same haplotype but never with F1534C or F1534L. In such a population, a single recombination event may lead to the production of haplotype PGC or PGL, which may have a greater impact on the insecticide resistance phenotype. Unlike our finding, a small proportion of mosquitoes in Myanmar have been found with homozygous F1534C + V1016G (2.9%, double mutant) and with homozygous S989P + V1016G + F1534C (0.98%, triple mutant) suggesting the presence of the PGC haplotype [47]. The occurrence of such a haplotype with three mutations is expected with a single recombination event, which may be selected in the presence of insecticide pressure and may lead to a higher degree of resistance. Such a combination has already been reported in Indonesia, where 21% of the population had the triple mutant [48].

In the present study, we always found V1016G present with S989P and *vice versa*. A similar association has been shown in a few other studies [39, 49]. However, in some studies, the frequency of V1016G has been reported to be higher than S989P, but S989P was always linked with V1016G [45, 47, 50]. A similar unidirectional linkage has been shown in domain III in VGSC of *Ae. aegypti* population in the Delhi-population, where a novel mutation T1520I was always found with F1534C and was suggested to be a compensatory mutation [15]. Previously, in expression studies carried out in *Xenopus* oocytes, Chen et al. [27] found that T1520I alone did not alter the VGSC sensitivity to permethrin or deltamethrin but had an additive effect to F1534C in protection against permethrin.

An unusual fact we recorded in this study was the non-compliance of HWE for F1534-*kdr* alleles in the Bengaluru population. A similar departure from HWE for this locus was also noted in our previous study carried out in Delhi (India) [15] and in Grand Cayman Island [44]. It was interesting to note that in the Delhi population [15], mentioned above, alleles at residue T1520 (Thr/Ile) complied with HWE. Currently, we do not have a definite explanation for this departure from the HWE; it may possibly be due to the presence of duplicated VGSC genes as proposed by Martin et al. [51]; however, this needs further investigation.

## Conclusions

To the best of our knowledge, this study reports for the first time the presence of a F1534L mutation in an *Ae. aegypti* population, which is associated with pyrethroid resistance alongside the presence of three other mutations *viz* F1534C, S989P and V1016G. Molecular methods were developed for monitoring of all these *kdr* mutations.

## Supplementary information

**Additional file 1: Table S1.** Frequency of individuals with different genotype combinations at residues F1534, S989 and V1016.

**Additional file 2: Text S1.** Estimation of gametic phase from multi-locus diploid data based on a Gibbs sampling strategy.

## Data Availability

All data generated or analysed during this study are included in this published article and its additional files.

## Abbreviations

ASPCR: allele-specific polymerase chain reaction
CI: confidence interval
DDT: dichloro-diphenyl-trichloroethane
DEL: deltamethrin
HWE: Hardy–Weinberg equilibrium
*kdr*: knockdown resistance
LLIN: long-lasting insecticidal net
OR: odds ratio
PCR-RFLP: polymerase chain reaction-restriction fragment length polymorphism
PCR: polymerase chain reaction
PER: permethrin
VGSC: voltage-gated sodium channel
