# Supplementary material for: A new knockdown resistance (kdr) mutation, F1534L, in the voltage-gated sodium channel of Aedes aegypti, co-occurring with F1534C, S989P and V1016G
Source: Parasit Vectors. 2020 Jun 29;13:327. doi: 10.1186/s13071-020-04201-3 (PMC7325290; doi:10.1186/s13071-020-04201-3)
Supplement: Supplementary file 1 — Additional file 1: Table S1. Frequency of individuals with different genotype combinations at residues F1534, S989 and V1016. [file 13071_2020_4201_MOESM1_ESM.pdf]

Additional file 1: Table S1. Frequency of individuals with different genotype combinations at residues F1534, S989 and V1016.

Genotype*			N (frequency)
F1534	S989	V1016	
FF	SS	VV	29 (0.04)
FC	SS	VV	147 (0.18)
FL	SS	VV	37 (0.05)
CC	SS	VV	216 (0.27)
LL	SS	VV	17 (0.02)
LC	SS	VV	157 (0.19)
FF	SP	VG	45 (0.06)
FC	SP	VG	108 (0.13)
FL	SP	VG	29 (0.04)
CC	SP	VG	0
LL	SP	VG	0
LC	SP	VG	0
FF	PP	GG	29 (0.04)
FC	PP	GG	0
FL	PP	GG	0
CC	PP	GG	0
LL	PP	GG	0
LC	PP	GG	0

*All samples were homozygous (TT) at residue T1520
