# Supplementary material for: A new knockdown resistance (kdr) mutation, F1534L, in the voltage-gated sodium channel of Aedes aegypti, co-occurring with F1534C, S989P and V1016G
Source: Parasit Vectors. 2020 Jun 29;13:327. doi: 10.1186/s13071-020-04201-3 (PMC7325290; doi:10.1186/s13071-020-04201-3)
Supplement: Supplementary file 2 — Additional file 2: Text S1. Estimation of gametic phase from multi-locus diploid data based on a Gibbs sampling strategy. [file 13071_2020_4201_MOESM2_ESM.pdf]

Additional file 2: Text S1. Estimation of gametic phase from multi-locus diploid data based on a Gibbs sampling strategy.

```
Number of individuals                : 814
Number of ambiguous individuals      : 182
Number of loci                       : 3
Number of polymorphic loci           : 3

List of estimated haplotype frequencies
---------------------------------------

[Hapl. ID]     Hapl. freq.    Haplotype definition
=========      =========      ==================
[     1]       287 (0.176)         S V F
[     2]       844 (0.518)         S V C
[     3]       257 (0.158)         S V L
[     4]       240 (0.147)         P G F
```
